# Methods for determining disease burden and calibrating national surveillance data in the United Kingdom: the second study of infectious intestinal disease in the community (IID2 study)

**DOI:** 10.1186/1471-2288-10-39

**Published:** 2010-05-05

**Authors:** Sarah J O'Brien, Greta Rait, Paul R Hunter, James J Gray, Frederick J Bolton, David S Tompkins, Jim McLauchlin, Louise H Letley, Goutam K Adak, John M Cowden, Meirion R Evans, Keith R Neal, Gillian E Smith, Brian Smyth, Clarence C Tam, Laura C Rodrigues

**Affiliations:** 1 School of Translational Medicine, University of Manchester, Manchester Academic Health Science Centre, Stopford Building, Oxford Road, Manchester, M13 9PL, UK; 2 MRC General Practice Research Framework, Medical Research Council General Practice Research Framework, Stephenson House, 158-160 North Gower Street, London, NW1 2ND, UK; 3 School of Medicine, Health Policy and Practice, University of East Anglia, Norwich NR4 7TJ, UK; 4 Centre for Infections, Health Protection Agency, 61 Colindale Avenue, London, NW9 5EQ, UK; 5 Regional Microbiology Network, Health Protection Agency, HPA North West Manchester Laboratory, Oxford Road, Manchester, M13 9WL, UK; 6 Regional Microbiology Network, Health Protection Agency Yorkshire and the Humber, Leeds Laboratory, York Road, Leeds LS15 7TR, UK; 7 Section of Public Health & Health Policy, University of Glasgow, 1 Lilybank Gardens, Glasgow, G12 8RZ, UK; 8 Department of Primary Care and Public Health, Cardiff University, Temple of Peace and Health, Cathays Park, Cardiff CF10 3NW, UK; 9 Department of Epidemiology and Biomedical Research Unit (Gastro), University of Nottingham, Nottingham, NG7 2UH, UK; 10 Health Protection Agency Real-time Syndromic Surveillance Team, Health Protection Agency, 6th Floor, 5 St Philip's Place, Birmingham, B3 2PW, UK; 11 Health Protection Service Northern Ireland, Public Health Agency, McBrien Building, Belfast City Hospital, Belfast, BT9 7AB, UK; 12 Department of Epidemiology and Population Health, London School of Hygiene and Tropical Medicine, Keppel Street, London WC1E 7HT, UK

## Abstract

**Background:**

Infectious intestinal disease (IID), usually presenting as diarrhoea and vomiting, is frequently preventable. Though often mild and self-limiting, its commonness makes IID an important public health problem. In the mid 1990s around 1 in 5 people in England suffered from IID a year, costing around £0.75 billion. No routine information source describes the UK's current community burden of IID. We present here the methods for a study to determine rates and aetiology of IID in the community, presenting to primary care and recorded in national surveillance statistics. We will also outline methods to determine whether or not incidence has declined since the mid-1990s.

**Methods/design:**

The Second Study of Infectious Intestinal Disease in the Community (IID2 Study) comprises several separate but related studies. We use two methods to describe IID burden in the community - a retrospective telephone survey of self-reported illness and a prospective, all-age, population-based cohort study with weekly follow-up over a calendar year. Results from the two methods will be compared. To determine IID burden presenting to primary care we perform a prospective study of people presenting to their General Practitioner with symptoms of IID, in which we intervene in clinical and laboratory practice, and an audit of routine clinical and laboratory practice in primary care. We determine aetiology of IID using molecular methods for a wide range of gastrointestinal pathogens, in addition to conventional diagnostic microbiological techniques, and characterise isolates further through reference typing. Finally, we combine all our results to calibrate national surveillance data.

**Discussion:**

Researchers disagree about the best method(s) to ascertain disease burden. Our study will allow an evaluation of methods to determine the community burden of IID by comparing the different approaches to estimate IID incidence in its linked components.

## Background

**Figure 1:**
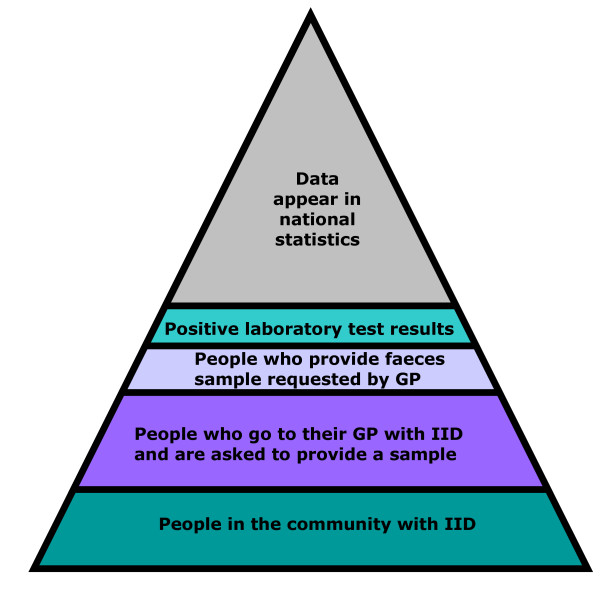
**The surveillance pyramid illustrating attrition of data at each level**.

Infectious intestinal disease (IID), usually presenting as diarrhoea and vomiting, is frequently preventable. Its commonness makes it an important public health problem. In the mid 1990s around 1 in 5 people in England were found to suffer from IID, with around 35,000 hospital admissions and 300 deaths annually, an estimated annual cost to the nation of £0.75 billion [1]. Although IID is very common in the community, not all cases present to the healthcare system, and not all cases that present are reported to national surveillance. Many IID cases are therefore not captured by routine data sources, a pattern described as the surveillance pyramid (Figure 1). Routinely available surveillance data in the United Kingdom, and elsewhere, thus underestimate the total IID burden, and that degree of underestimation needs to be calibrated [1].

Most studies for estimating community disease burden in developed countries are either prospective cohort studies or retrospective cross-sectional surveys. The prospective cohort design consists of recruiting volunteers and asking them to record relevant symptoms, often in some form of diary. The retrospective study involves contacting people, usually by telephone and asking about symptoms in the recent past. A major advantage of population-based, prospective cohort studies is the ability to request stool specimens from people who report illness so that the range of gastrointestinal pathogens causing symptoms can be determined. Retrospective study designs provide no information on the microbiological causes of illness (a major weakness) but are significantly quicker and less costly to complete.

Our present study builds on a previous study of IID conducted in England during the early 1990s, which underlined the public health impact of gastrointestinal infection [1]. At that time, approximately 20% of the population was found to suffer from IID annually.

The only other notable prospective study to date, based on active detection of IID, was conducted in the Netherlands. Whilst in England there were an estimated 194 cases of infectious intestinal disease (IID) per 1,000 person years (equivalent to 0.2 per person per year) [1], in the Netherlands there were 283 cases per 1000 person years (equivalent to 0.3 per person per year) [2]. Although the two studies shared some common methods, the case definitions differed.

A major contribution of the IID study in England was calibration of the national surveillance system, i.e. estimating the factor by which the reported number of infections with specified pathogens needed to be multiplied in order to establish the actual number of infections in the community. For every case of IID reported to national surveillance, 136 cases had occurred in the community. For campylobacters it was estimated that the ratio of cases in the community to those reported to national surveillance was 8 to 1. For salmonellas and noroviruses the corresponding ratios were 3 to 1 and over 1,500 to 1 respectively [1].

By contrast there are many more retrospective studies reported in the literature, mainly from North America [3]. These retrospective studies have been used in both outbreak and non-outbreak settings [4-8]. The studies have varied in their estimation of illness rates from 0.6 to 3.2 episodes per person year, though case definitions often differed from one study to another. For example, in the US, the overall prevalence of self-reported acute diarrhoea in the four weeks before telephone interview in randomly selected residents from defined catchment areas was 11% or 1.4 episodes of diarrhoea per person per year [6]. In Australia, in a cross-sectional telephone survey of randomly selected individuals, 11.2% of respondents reported gastroenteritis with an overall weighted incidence of 0.92 (95%CI 0.77-1.6) cases per person per year [9].

The problem is that estimates of disease burden in the community differ substantially between retrospective and prospective study designs even when using identical case definitions. This was highlighted in original the IID Study where the retrospective element of the study estimated disease incidence at 0.55 episodes per person per year and the prospective element at 0.19 [10]. At the time this discrepancy was attributed to "telescoping" whereby people remember disease episodes as being more recent than they actually are, thereby inflating the incidence over the recall period. Further concerns about the validity of retrospective surveys of diarrhoeal incidence were raised in a study centred on a waterborne outbreak [11].

However, a number of explanations, in addition to telescoping, might explain the discrepancy in incidence rates derived from prospective and retrospective studies. Potential issues with active reporting in population-based prospective cohort studies include loss to follow-up, sensitisation and reporting fatigue. In the IID Study in England 39% of the original cohort of 9,296 were lost to follow-up over 6 months and it is not clear whether loss to follow-up might be correlated with risk of illness. Sensitisation occurs when respondents become more aware of their health because they are participating in a health-related study [12], and may perceive more symptoms during early follow-up than before enrollment. If they subsequently become fatigued with completing a health diary, or returning data via postcard or e-mail, the number of symptoms reported over time may decline [12,13]. This pattern of sensitisation-fatigue, where illness reporting is highest during the early weeks of follow-up and subsequently decreases, is characteristic of much longitudinal data [12,14,15]. Fatigue might also have played a part in the decline in data quality observed in some studies [14,16-18]. Sensitisation and fatigue are potential problems because estimated incidence rates may vary as a function of the length of the follow-up period if either of these time-dependent conditioning effects is present [12].

For retrospective studies the main problem is accuracy of retrospective reporting. This includes not only inaccuracy in the respondent remembering whether or not they suffered the symptoms requested but whether they recall other important information such as the date of onset of symptoms, duration and severity. The potential issue of telescoping, whereby people report illness that occurred just outside the recall period as having occurred during the recall period, so increasing the estimated disease incidence, has already been raised. Conversely people might be more likely to remember events that occurred close to the interview, so reducing the estimated incidence.

A further issue with both study designs is the issue of the representativeness of participants. Prospective studies can be designed to invite a representative sample of the population according to whatever criteria the investigators choose. However, in most studies a relatively small proportion of those invited is actually recruited and it is uncertain whether the symptoms under investigation differ between those who accept and decline the invitation. By contrast telephone surveys are much more difficult to target at a representative sample of the community, though response rates are usually higher. Particular issues of concern are the increasing proportion of homes without access to a landline, especially amongst young adults and people with jobs or lifestyles that mean they are away from the home during times when most calls tend to be made.

Another of the major challenges in international comparisons of IID incidence rates, regardless of the study design, is the variation in case definitions. Indeed the case definition can influence the observed incidence of gastroenteritis by as much as 1.5-2.1 times in a given country [19]. To overcome this, a standard, symptom-based definition has been developed that should allow international comparison in the future [19]. We have included information in our clinical questionnaire that will allow us to report rates according to the international definition for both the telephone survey and the prospective cohort study.

### Research Questions

During the past decade in the UK, reported rates of laboratory-confirmed infections associated with IID appear to have fallen. However, this may not reflect a true decline in disease as there have been structural changes that could affect national surveillance over the same time period. In primary care, people seeking advice about IID can now contact NHS Direct (or NHS24 in Scotland), a 24-hour, nurse-led, telephone helpline rather than consult their general practitioner (GP). Clinical laboratories no longer report directly to the national centre in England but via regional units. The creation of the Health Protection Agency in 2003 reduced from 48 to nine the number of lead laboratories directly under the control of the public health services with a possible reduction in the range of microbiological tests applied to each specimen. Conversely, there have been huge advances in the methods available to detect gastrointestinal pathogens, notably the introduction of molecular methods. It is therefore unclear whether the reported decline in IID is due to a genuine reduction in incidence. Answering this question is important in order to direct future food safety policy.

The principal research question in the Second Study of Infectious Intestinal Disease in the Community (IID2 Study) is:-

• Has the incidence of IID in England in the community declined since the mid 1990s?

The secondary research questions are:-

• What is the aetiology of IID in the community, and presenting to GPs, and by how much do national surveillance data in the UK underestimate the community and GP burden of IID?

• How much IID is acquired abroad?

• How do molecular methods compare with traditional microbiological methods for IID diagnosis?

• What is the best research method for determining IID incidence in the community?

## Methods/Design

**Figure 2:**
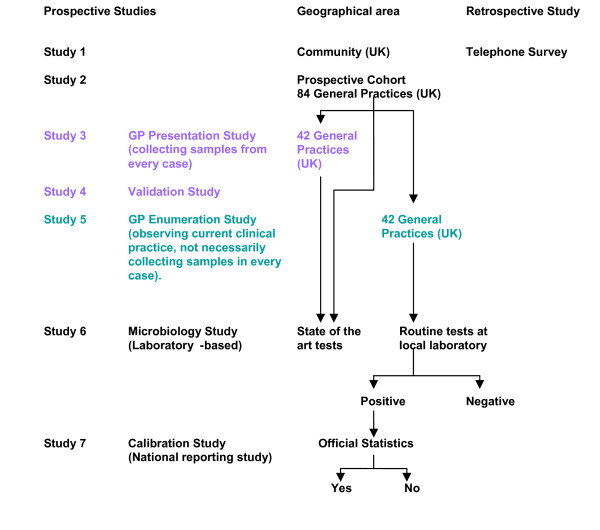
**The structure of the IID2 Study**.

The IID2 study comprises seven separate but related studies (Figure 2). All studies take place simultaneously over a period of 12 months.

### Case definition used in all studies

Cases are defined as persons with loose stools or clinically significant vomiting lasting less than two weeks, in the absence of a known non-infectious cause, preceded by a symptom-free period of three weeks. Vomiting is considered clinically significant if it occurs more than once in a 24-hour period and if it incapacitates the case or is accompanied by other symptoms such as cramps or fever.

### Exclusion criteria used in all studies

The exclusion criteria are:-

• Patients with terminal illness.

• Patients whose first language was not English and for whom a suitable interpreter was not available.

• Patients with severe mental incapacity.

• Patients with non-infectious causes of diarrhoea: Crohn's disease, ulcerative colitis, cystic fibrosis, coeliac disease, surgical obstruction, excess alcohol, morning sickness, regurgitation in infants. The reason for these exclusions is because it is difficult to determine dates of onset for acute IID in the presence of chronic bowel disease.

### Participant identification and recruitment

The first study is a UK-wide, all-age, **telephone survey of self-reported IID**. It is a community-based, retrospective survey with a target recruitment of 14,400 respondents. Recruitment is via a two-stage sampling process. First, households are selected at random from the populations of the four countries of the United Kingdom through a process of random digit-dialling of landlines. Second, in homes where there is more than one family member who could potentially take part, the participant is selected at random by asking the person who answers the telephone to invite the person in the household whose birthday occurs next to take part in the study. The telephone survey takes place on weekday evenings, between 18:00 and 21:00 or on Saturday/Sunday between 14:00 and 17:00. Each number is 'phoned three times (including at least once at the weekend and once during the week) before being deemed non-contactable. Respondents aged = 12 years are interviewed directly, and a parent or guardian is interviewed about participants aged < 12 years. Oral informed consent is obtained from all participants and parents of children aged < 16 years. The telephonists administer a standard questionnaire that includes clinical history, history of recent foreign travel and any clinical consultations, including those with NHS Direct (NHSD) or NHS24 (in Scotland). All calls are recorded using "CopyCall" software. To investigate whether accuracy of symptom reporting varies according to the recall period, we assign participants randomly to be questioned about symptoms occurring either within the previous seven days or 28 days.

The second study is a **prospective, all-age, population-based cohort study of self-reported IID**. The sampling frame is all 912 general practices in the UK that are part of the Medical Research Council General Practice Research Framework http://www.gprf.mrc.ac.uk/. We mail study details to all the 912 practices to seek expressions of interest. A minimum of 84 practices is required (see section on Sample Sizes below). Trained nurses in each participating practice generate a list of 800 potential participants at random from the practice list with a view to recruiting at least 100 participants per practice. This is scrutinised by the general practitioner (GP) to ensure that it is appropriate to contact the individuals. For example, the GP checks that the list does not contain the name of someone who is very frail or recently deceased.

The GP sends a letter to potential participants containing an invitation to take part and an information sheet which explains the study and what is involved if they agree. We ask participants to return a reply-paid slip to their GP surgery indicating whether or not they wish to discuss the study further with the practice research nurse. If so, the nurse contacts them to arrange a suitable appointment, explains the study in greater detail and, if appropriate, obtains written consent. The participant also completes a very short, baseline questionnaire requesting basic demographic details of age, sex, address (including post code) and occupation.

Participants are followed up weekly for one calendar year from the date of recruitment to find out if they have any episodes of IID. Follow-up is either by secure, automated e-mail or by weekly postcard, depending on the participant's preference, and employs negative reporting; participants are asked to reply each week even if they have had no symptoms. Each participant is provided with a faecal specimen kit (specimen pot, instructions on how to obtain a specimen, reply-paid, Post Office approved packaging), and a short symptom questionnaire with reply-paid envelope.

Participants are reminded that, if they develop symptoms, they should send a sample promptly to the Health Protection Agency Regional Laboratory in Manchester. They also complete the symptom questionnaire, which they return to the study team in the reply-paid envelope. The symptom questionnaire asks about date of onset and duration of symptoms, symptom profile and severity, healthcare-seeking behaviour (including contact with NHS Direct or NHS24, contact with or visits to the primary care team or visits to hospital and any overnight stays) and history of foreign travel in the ten days before symptom onset.

The third study is a **prospective study of all patients presenting to their GP with symptoms of IID (GP Presentation Study)**. This takes place in half the practices that are undertaking the prospective cohort study. Anyone registered in the practice who consults their GP for an episode of IID is eligible to take part, unless disallowed by the exclusion criteria (see above). The GP identifies eligible patients and refers them to the practice research nurse, who explains the study. Patients that agree to participate complete a consent form, and are given a specimen kit (as described above), and a symptom questionnaire with reply-paid envelope. The participant provides the sample and completes the questionnaire at home, posting them back to the study team using the reply-paid envelopes.

The fourth study is **an audit of the IID2 study recruitment process (GP Validation Study)**. Using a standardised, pre-defined set of diagnostic codes (Read codes [20]) the research nurse searches the practice database for every patient that presents with IID during the study period. They cross-reference this list with the cases recruited to the GP Presentation Study to determine the proportion of eligible participants that are not recruited. This will allow adjustment for under-ascertainment at the end of the study.

The fifth study is a **survey of routine clinical practice for IID and of organisms identified in IID in routine laboratory practice (GP Enumeration Study)**. It is conducted in the practices not undertaking the GP Presentation Study. The practice research nurse identifies from the practice database all patients who present with a new episode of IID during the study period. They record the case's age, sex, postcode, and information about the place of consultation, admission to hospital and whether or not a faecal specimen has been requested. If a sample was requested as part of the consultation the nurse records the result. By comparing the results of the GP Presentation and Enumeration Studies we will determine the relationship between the total number of people who consult their GP with IID and the number of people who consult with IID and have their infection laboratory confirmed in routine clinical practice (which we anticipate will be smaller).

The **laboratory-based studies **take place at the Health Protection Agency (HPA) Regional Laboratory in Manchester and the HPA Centre for Infections in London. All faecal specimens from the prospective Cohort Study and the GP Presentation Study are examined first at the Manchester laboratory using conventional microbiological methods (Table 1).

**Table 1:** Target Organisms: Primary Diagnostic Methods.

Bacteria	Methods
*Campylobacter jejuni*/coli*	Direct plating - modified cefeoperazone, charcoal deoxycholate (CCD) agar. Enrichment culture - Preston broth.

*Clostridium perfringens *(enterotoxin)	Techlab™ (Blacksburg, USA) enzyme linked immunosorbent assay (ELISA), all positives to be cultured and isolates sent to the reference laboratory.

*Clostridium difficile *cytotoxin	Premier™ (Meridian Bioscience Inc., Cincinnati, OH) toxins A and B enzyme immunoassay (EIA)

*Escherichia coli *O157*	Direct plating on Cefixime Tellurite Sorbitol MacConkey agar. Enrichment in Modified Tryptone Soya Broth with Novobiocin.

*Listeria *spp (*monocytogenes*)*	Direct plating - polymyxin acriflavine lithium chloride ceftazidime asculin mannitol (PALCAM) agar**

*Salmonella *spp*	Direct plating - Xylose Lysine Dextrose (XLD) Agar and Desoxycholate Citrate Agar (DCA). Enrichment culture - Selenite F broth and Rappaport Vasilliades Salmonella enrichment broth.

*Shigella *spp*	Direct plating - XLD and DCA.

*Yersinia *spp*	Direct plating - Cefsulodin Irgasin Novobiocin (CIN) selective agar. Enrichment culture - Tris Buffer Yersinia enrichment broth.

**Protozoa**	

*Cryptosporidium parvum*	Techlab™ Giardia/Cryptosporidium check, r-biopharm™ RIDA™ Quick Cryptosporidium

*Giardia intestinalis*	Techlab™ Giardia/Cryptosporidium check, r-biopharm™ RIDA™ Quick Giardia

Cyclospora	Modified Ziehl-Neelsen (ZN) stain

**Viruses**	

Enteric viruses	(Premier™ Rotaclone, Premier™ Adenoclone).

* All positive isolates were sent to the relevant reference laboratory.

** PALCAM agar was used in previous studies [32,33]

Notes:

1. If the clinical information indicated a history of foreign travel then specimens were tested for *Vibrio *spp (Direct plating using Thiosulphate Citrate Bile Sucrose (TCBS) agar and alkaline peptone water (APW) enrichment) and ova, parasites and cysts (Direct and concentration microscopy)

2. If the specimen was related to an outbreak investigation then it was tested for other pathogens e.g. *Staphylococcus aureus *(Direct plating MSA), *Bacillus *spp. (direct plating Bacillus cereus agar - MEYP).

3. Specimens from children < 5 years old were examined for Rotavirus and Adenovirus 40,41 by immunoassay (Premier™ Rotaclone, Premier™ Adenoclone).

All specimens are tested on the day of receipt. An initial 10% suspension of the stool specimen is made in 0.1% peptone water and used to inoculate the various selective plating media and enrichment broths. At this stage the specimens are cultured for *Campylobacter jejuni/coli*, *Escherichia coli *O157, *Listeria monocytogenes*, *Salmonella *species, *Shigella *species and *Yersinia *species. They are also examined by enzyme-linked immunoassay (EIA) for *Clostridium perfringens *enterotoxin, *Clostridium difficile *toxins A and B, *Cryptosporidium *and *Giardia *and by light microscopy for *Cyclospora*.

We use two approaches for detecting viruses. Specimens from children <5 years old are examined for Rotavirus and Adenovirus 40, 41 by immunoassay. This is routine clinical practice, which supports clinical management of the participants. All samples are then examined for the five major viral pathogens by quantitative polymerase chain reaction, which has meant developing suitable methods for assigning positive results based on cycle threshold values [21,22].

As part of the routine diagnostic algorithm, specimens from patients with a history of foreign travel are also tested for *Vibrio *spp. and for the ova and cysts of parasites using National Standard Methods (BSOP30 and BSOP31 (available at http://www.hpa-standardmethods.org.uk/national_sops.asp)). If the patient is considered to be part of a potential food poisoning outbreak the specimens are cultured for *Clostridium perfringens, Staphylococcus aureus *and *Bacillus *species using National Standard Methods (BSOP30). All isolates of the major enteric bacteriological pathogens are submitted to the HPA Centre for Infections for strain characterisation.

Specimens are batched and sent from Manchester to the HPA Centre for Infections by courier twice a week. Two nucleic acid extracts are prepared from each sample of faeces by a modification of the method of Boom and colleagues [23]. For one sample (DNA) mechanical disruption using zirconia beads is included [24], and in the second, RNA is immediately converted to cDNA through random primed reverse transcription [25]. The reverse transcriptase reactions using random hexamer priming have been described elsewhere [26-28]. Each extract is examined by real-time PCR for a range of potential pathogens (Table 2). These are *Campylobacter jejuni, Campylobacter coli*, *Clostridium perfringens*, *Clostridium difficile*, *Listeria monocytogenes*, *Salmonella *species, rotavirus, norovirus, sapovirus, adenovirus, astrovirus, *Cryptosporidium*, *Giardia *and *E. coli *(enteroaggregative and verocytotoxigenic (genes encoding VT1 and VT2)). A PCR result is considered to be positive if the cycle threshold value (CT value) is less than 40. Specimens that are PCR-positive for *C. difficile *toxin are cultured and the isolates are ribotyped.

**Table 2:** Assays used to detect a range of bacterial, viral and parasitic pathogens.

PCR (SOP)	Assay - chemistry	Target Organism	Gene encoding proteins	References
NOR1	SINGLE-5'exonuclease	Norovirus genogroup 1	RNA dependent RNA polyermerase/capsid	[34]

NOR2	DUPLEX-5'exonuclease	Norovirus genogroup 2 Mengo virus mutant vaccine strain MC	RNA dependent RNA polyermerase/capsid Not known	[35] Comite Europeen de Normalisation (CEN)

ROTA	SINGLE-5'exonuclease	Rotavirus Group A	Viral Protein 6	[35,36]

SAPO	DUPLEX-5'exonuclease	Sapovirus	Polymerase-capsid junction (2 probes)	[37]

ASTR	SINGLE-SYBR Green	Astrovirus	Capsid	[38]

ADEN	SINGLE-5'exonuclease	Adenovirus type 40 and 41	Long fibre protein	[39]

CAMP	DUPLEX-5'exonuclease	*C. jejuni* *C. coli*	Membrane associated protein Lipoprotein of iron binding protein	[40]

SALM	DUPLEX-5'exonuclease	*Salmonella enterica* Green Fluorescent Protein gene (*gfp*) inserted into a *E. coli*	Glycotransferase GFP Protein	[41]

EAGG	SINGLE-5'exonuclease	EnteroAggregative *E. coli*	Anti aggregation transporter	[28,42]

LIST	SINGLE-5'exonuclease	*Listeria monocytogenes*	Haemolysin A	[43]

VT1-VT2	DUPLEX-5'exonuclease	Verocytotoxin 1 Verocytotoxin 2	Verocytotoxin 1 Verocytotoxin 2	[44]

GIAR	SINGLE-5'exonuclease	*Giardia *spp.	Elongation Factor 1 alpha	[43]

CRYP	DUPLEX-5'exonuclease	*C. hominis, C. parvum, C. meleagridis, C. felis*	*Cryptosporidium *oocyst wall protein	[43]

CPER	DUPLEX-5'exonuclease	*C. perfringens *alphatoxin and enterotoxin	Phospholipase C gene of *C. perfringens* Enterotoxin gene of enterotoxigenic *C. perfringens*	[45]

CDIF	MULTIPLEX- 5'exonuclease	Toxin-producing *C. difficile*	Toxin B gene (*tcdB*), binary toxin (*cdt*), and *tcdC *gene single-base deletion at nucleotide 117 (*tcdB*)	[46-48]

Two samples of 1-2ml each of a 10% faecal suspension, 5 × 10 μl of a DNA extract and 5 × 10 μl of cDNA extract are archived for future study. Participants in the study give their explicit consent for this.

Finally, in the **National Reporting Study**, we calibrate national surveillance data to determine the relationship between disease burden in the community and infections reported to national surveillance. To do this we correlate reports of laboratory-confirmed IID due to the target pathogens in the IID2 Study firstly with self-reported IID in the Cohort Study, secondly with telephone calls to NHS Direct or NHS 24 for people reporting IID, and thirdly with patients consulting for IID in the GP Presentation Study. Each of the studies described above provides a slice through the surveillance pyramid (Figure 1). By comparing rates and aetiology at each level of the pyramid we can determine by how much national surveillance data underestimate IID burden in the community.

### Sample Sizes

The sample size calculations for the telephone survey, prospective cohort study and GP Presentation Study are presented in Tables 3, 4, and 5 respectively. The assumptions used in the sample size calculations are presented in each table.

**Table 3:** Sample size calculations for estimating the overall frequency of IID via self-report telephone survey.

Duration of recall period	Incidence in original IID recall Questionnaire	Widest acceptable CI limit	Number needed to survey
28 days	6%	4%	500

7 days	1.5%	1%	2500

Note: Based on an expected frequency of IID of 6%, with a 95% Confidence Interval (CI) of 4% to 8%. Allowing for differentials in response rate the number needed to survey was increased by 20% i.e. to 600 for recall over one month and 3000 for recall over one week.

**Table 4:** Sample size calculations for prospective cohort study estimating a single UK-wide pyramid.

	England				Wales	
**Organism/condition**	**Baseline incidence**^1^	**Reduction to be detected**	**Person-years**	**GP practices**	**Person-years**	**GP practices**

All IID	19.20%	20%	2,000	20	200	2

Severe cases*	6.00%	20%	7,000	70	400	4

*Campylobacter*	0.87%	20%	500,000	5,000	2,400	24

*Salmonella*	0.22%	20%	500,000	5,000	9,500	95

*Campylobacter*+*Salmonella*	1.10%	20%	200,000	2,000	2,000	20

*Campylobacter*+*Salmonella+ C. perfringens*	1.34%	20%	100,000	1,000	1,600	16

	**Scotland**		**Northern Ireland**		**UK**	

**Organism/condition**	**Person-years**	**GP practices**	**Person-years**	**GP practices**	**Person-years**	**GP practices**

All IID	200	2	65	1	2,465	25

Severe cases*	700	7	300	3	8,400	84

*Campylobacter*	4,200	42	1,400	14	508,000	508

*Salmonella*	16,400	164	5,500	55	531,400	532

*Campylobacter*+*Salmonella*	3,400	34	1,200	12	206,600	207

*Campylobacter*+*Salmonella+ C. perfringens*	2,800	28	1,000	10	106,200	107

* Severe cases are defined as those presenting to primary care

Note: Based on ability to detect a 20% decrease in incidence with 80% power and 95% precision. Required number of person-years and GP practices (recruiting 100 patients from each practice) by country, based on the relative populations of the 4 UK countries

**Table 5:** Sample size estimates for GP Presentation Study estimating a single UK-wide pyramid.

	England				Wales	
**Organism**	Baseline incidence*	**Reduction to be detected**	**Person-years**	**GP practices**	**Person-years**	**GP practices**

Campylobacter	4.10%	20%	115,000	20	7,000	2

*Salmonella*	0.16%	50%	41,000	7	3,000	1

*Salmonella*	0.16%	40%	67,000	12	4,000	1

*Salmonella*	0.16%	30%	127,000	22	8,000	2

*Salmonella*	0.16%	20%	302,000	51	18,000	3

*C. perfringens*	0.13%	20%	364,000	61	22,000	4

	**Scotland**		**Northern Ireland**		**UK**	

**Organism**	**Person-years**	**GP practices**	**Person-years**	**GP practices**	**Person-years**	**GP practices**

Campylobacter	12,000	2	4,000	1	138,000	25

*Salmonella*	5,000	1	2,000	1	51,000	10

*Salmonella*	7,000	2	3,500	1	81,500	16

*Salmonella*	13,000	3	4,500	1	152,500	28

*Salmonella*	31,000	6	10,500	2	361,500	62

*C. perfringens*	38,000	7	13,000	3	434,500	75

* Incidence of GP presentation in original IID study

Note: Based on the ability to detect at least a 20% reduction in incidence with 90% power and 95% precision. Required number of person-years and GP practices (assuming an average GP practice size of 6,000 patients) by country, based on the relative populations of the 4 countries.

### Plan of Analysis

The proposed analytical strategy is as follows:-

*Methods for assessing representativeness, response bias, compliance and list inflation for the prospective study and the telephone survey*.

#### Representativeness

Representativeness of the Cohort Study and Telephone Survey sample will be assessed by comparing characteristics of the respective study populations with routine National Census data on age distribution, sex, ethnic group, urban-rural residency, socioeconomic group, and area-level deprivation. For the GP Presentation Study, representativeness of the population of participating practices will be assessed by comparing the age and sex distribution of the registered population with that of the census.

#### Compliance

Compliance in the Cohort and GP Presentation Studies will be calculated as the proportion of participants reporting symptoms who submit a questionnaire and stool sample. Differences in compliance across age, sex and socioeconomic group will be assessed with the Pearson chi-square test and multivariable logistic regression.

#### Under-ascertainment

Factors influencing under-ascertainment in the GP Presentation Study will be investigated using multivariable logistic regression. The inverse of the predicted under-ascertainment from this model will be used as a multiplier for the number of cases identified in the GP Presentation Study.

#### Overall incidence of IID

a. The overall incidence rate of IID in the community will be calculated from the total number of cases reported in the Cohort Study and the follow-up time of members of the cohort.

b. The overall incidence rate of IID presenting to general practice will be calculated from the total number of cases reported in the GP Presentation Study, adjusted for under-ascertainment, and in the Enumeration Study, and the follow-up time of all persons registered in the practice populations. Adjustments will be made to the denominator (practice list size) of the GP presentation rates to adjust for list inflation.

c. For the Telephone Survey, annualised rates of IID in the community, presenting to general practice and contacting NHS Direct/NHS 24 will be estimated separately for one month and one week recall periods, after adjustment for seasonality, separately for each of the four countries, and for the UK overall.

All incidence estimates will be standardized to the UK census population to enable comparison.

#### Prevalence of aetiological organisms in IID cases and pathogen-specific incidence

The prevalence of each pathogen among IID cases will be estimated by calculating the proportion of specimens tested that are positive for that organism. This will be done separately for the Cohort and GP Presentation studies.

For organisms for which both conventional and molecular diagnostic methods are used, the performance of the two methods will be compared.

Pathogen-specific prevalences will be used to determine the number of cases positive for that organism expected in the Cohort and GP Presentation studies, adjusted for non-compliance in submitting a stool specimen and under-ascertainment. Adjustments will be made assuming that the observed distribution of organisms is representative of all IID cases. These adjusted numerators will then be used to estimate the incidence of IID for each pathogen, both in the community and presenting to general practice. Confidence intervals will be calculated by a method that does not increase the apparent precision due to this adjustment.

#### Clustering

Practice-level variation in rates will be analysed using Poisson regression. Over-dispersion in GP presentation rates will be modelled with a random effects term at the practice level. Any observed clustering effect will be incorporated into all confidence intervals applied to GP presentation rates. Analysis will be based on episodes (IID symptoms separated by a symptom-free period of three weeks) rather than individuals, assuming each episode to be independent.

Reporting pyramids will be calculated from the overall incidence rates in the community and presenting to GPs by considering the ratios between these rates. Performing the same calculation on the upper and lower confidence intervals for the rate at each stage will derive the upper and lower sensitivity bands. The ratio of community rates to nationally reported cases will be calculated by projecting the overall cohort and presentation incidence rates to the population and comparing these to that reported to the laboratory reporting surveillance system over the same period. When more than one laboratory method was used, these will be estimated separately for results from both methods, for comparison with the first IID Study and as a baseline for the future.

#### Comparison of incidence in the Prospective and Telephone Survey studies

The incidences of IID in the community and presenting to general practice from the prospective studies will be compared with those from the Telephone Survey (using one month and one week recall). Incidence ratios will be estimated for each age, sex and recall period.

### Ethical approval to perform the IID2 Study

A favourable ethical opinion to perform the whole of the study was granted by the North West Research Ethics Committee (07/MRE08/5). Research management and governance was then sought, and approval granted by all relevant Research and Development Organisations across the United Kingdom.

## Discussion

Various aspects of the study design merit discussion. First, in the Telephone Survey we have elected to use landlines as the means of recruiting participants. Although fixed telephony has declined from a peak of 93% of households in 2003, some 87% of households still had a fixed line phone in the first quarter of 2009 (Ofcom, 2009 http://www.ofcom.org.uk/research/cm/cmr09/cmr09.pdf). This compares with 92% of homes that own one or more mobile phones. Much of this increase in mobile telephony is thought to reflect an increase in mobile phone ownership amongst children in previously non-mobile owning households. The issue of the impact of increasing use of mobile phones on epidemiological surveys is a difficult one. The risk of introducing bias by not using mobile phone numbers is offset by a number of considerations:-

• the use of mobile phone numbers in such surveys has not yet become standard and reliable sampling frames for these are not easily available

• many mobile phone users are children and we cannot contact children directly for ethical reasons

• it is not easy to localise mobile phones to a geographical area.

In general terms, people without fixed line phones tend to be younger and of lower socio-economic status - groups who tend not to respond well to surveys. It is thus unclear whether use of mobile phone numbers would help to mitigate selection bias [29]. However, to assess the potential for bias introduced by only using landlines, people recruited into the Cohort Study are asked about their main method of telephony. Furthermore, we shall assess the representativeness of the participants in the Telephone Survey compared with the general population.

Secondly, in the Cohort Study we are not collecting specimens from asymptomatic people in the community. This was, however, undertaken in the first IID study and provided very useful information on asymptomatic people carrying potential pathogens [10]. Using data only from IID cases to attribute illness to a particular organism can lead to an overestimation of prevalence, as some cases may be excreting organisms that are not directly responsible for their illness; comparing frequency of organisms in cases and controls is the best way of establishing the pathogenicity of an organism [10]. It has recently been shown that illness due to norovirus and rotavirus is closely related to viral load [21]. Using PCR based analysis and data from the first IID study, in which a nested case-control study was performed within the cohort study, we will be able to determine thresholds for symptomatic infection versus asymptomatic excretion using CT values [22]. The potential disadvantage of sending specimens unrefrigerated by conventional post is that labile organisms like *C. jejuni *may die off before the specimen reaches the laboratory. However, this is offset by the high sensitivity of using molecular methods in addition to conventional culture techniques. It is possible that, using molecular methods, we will find evidence of mixed infections, and indeed this was the case when PCR based techniques were used to re-examine specimens from the first IID study [28]. Using this approach may allow us to determine the contributions of those pathogens to patients' symptoms. In addition the major research question of "Has the incidence of IID in the community declined since the mid 1990s?" can be answered by a comparison of data from cases in this study with that from the first IID study.

Thirdly, we recognise that recruiting people to studies during a primary care consultation poses a number of challenges - not the least of which is asking the GP to remember the study in the midst of a busy clinic [30,31]. To address this we have developed posters and information leaflets for waiting rooms and consultation rooms, which give people who consult with IID symptoms the opportunity to ask about the study during the consultation. They also serve to remind practice staff that the study is taking place. We have also placed a referral pad in each consulting room so that, should the GP or nurse practitioner be too busy to take the patient through the study material at the time of the consultation, the patient can still be referred to the research nurse to receive information about the study. Despite these measures we recognise that not all eligible cases will be invited to take part. In the first IID study the records of 2,021 cases of possible IID were examined, 1514 (75%) fulfilled the case definition and 974 (64%) were included in the GP Presentation Study [10]. It is for this reason that we are auditing recruitment to the IID2 Study in the Validation Study. We will be able to adjust the final results, taking account of under-ascertainment of cases i.e. those who should have been recruited to the GP Presentation Study but were not.

Fourthly we have used a very sensitive case definition. This means that we may detect alterations in bowel habit that are not necessarily due to gastrointestinal pathogens, leading to a fairly large diagnostic gap (i.e. proportion of specimens negative for gastrointestinal pathogens). Alternatively, the case definition may be insensitive since it excludes subjects with diarrhoea of more than two weeks duration and may therefore exclude infections with some pathogens, particularly the protozoan parasites. However, we are using the same case definition as was employed in the first IID Study to allow us to compare rates between the two studies directly. We should also be able to report rates compatible with the proposed international case definition.

Finally, the opinions of researchers worldwide are divided over the optimal method(s) for undertaking community burden of IID studies. Rate estimates vary quite considerably, depending on which methods are used. To our knowledge, ours is the first study in which population-based prospective and retrospective studies are being conducted simultaneously. Therefore, in addition to fulfilling our primary objectives we will be able to provide an objective evaluation of methods for determining the community burden of IID in the future.

## Competing interests

The authors declare that they have no competing interests.

## Authors' contributions

SJOB, GR, PRH, JJG, FJB, JMcL, DST, CCT and LCR conceived the ideas for the study. All authors participated in its design and co-ordination and helped to draft the manuscript. SJOB, GR and LHL lead the prospective studies. PRH leads the telephone survey. JJG, FJB and DST lead the laboratory studies. GKA, JMC, MRE, KRN, GES and BS participate in the national surveillance studies. CCT and LCR lead the analytical strategy. All authors have read and approved the final manuscript.

## Pre-publication history

The pre-publication history for this paper can be accessed here:

http://www.biomedcentral.com/1471-2288/10/39/prepub
